# Infrequent Pediatric Subungual Injury Diagnosed by Intraoperative Anatomopathological Material: A Case Report

**DOI:** 10.7759/cureus.51482

**Published:** 2024-01-01

**Authors:** Alberto Daniel Navarro Vergara, Alberto Navarro Fretes, María Mercedes Medina Villate

**Affiliations:** 1 Orthopedics and Traumatology, Hospital de Trauma “Manuel Giagni”, Asunción, PRY; 2 Orthopedics and Traumatology, Hospital de Especialidades Quirúrgicas Ingavi del Instituto de Previsión Social, Asunción, PRY; 3 Orthopedics and Traumatology, Universidad del Norte, Asunción, PRY

**Keywords:** periungual edge impingement, onychogryphosis, hallux, benign bone tumor, subungual exostosis, tumor exeresis, infrequent subungual injury, female pediatric patient

## Abstract

Subungual lesions are very common in clinical practice. We present the clinical case of a 10-year-old female patient who presented with progressive nail deformity. The onset of the condition was approximately five years prior to presentation with an injury in the left hallux, according to the mother. She denied pain or change in the color of the area from the onset of the injury to the day of consultation. There was no previous trauma. Examination revealed subungual bone injury to the distal extremity (distal phalanx of the left hallux), and imaging tests (X-ray and soft tissue ultrasound) found bone injury. Subungual exostosis was considered as a possible diagnosis, thus prompting the indication for exeresis of the tumoral process. After surgical removal, the resected specimen was sent for pathological assessment, which found that an intraosseous hemangiolymphangioma was the origin of the tumor. A subungual exostosis is a slow-growth benign osseous tumor mainly located in the distal phalanx of the hallux that especially affects young adults, being less frequent in children. This condition results from a process of bone neoformation involving different stages, the clinical symptoms of which depend on its size and associated processes. Hemangiolymphangiomas are angiomatous lesions of the blood and lymphatic vessels that have a controversial etiology and present slow, painless, and progressive growth; these lesions are mostly benign. It is worth emphasizing that subungual injuries are not always caused by an underlying bone; therefore, potential differential diagnoses, both benign and malignant, should be considered, based on the location of the injury.

## Introduction

The barriers to preventing infection in healthy nails depend on the compact nail plate [1]. Each nail condition presents different characteristics [2].

The nail plate, nail matrix, hyponychium, nail bed, and surrounding nail folds constitute the nail unit [3]. In the pediatric population, most nail disorders generally have a favorable prognosis. The anxiety observed in patients and parents due to nail abnormalities is described knowing that most nail disorders in pediatric patients are benign and generally have a favorable prognosis [4].

Clinical evaluation in a pediatric patient with a nail disorder always is a challenge and needs to be taken carefully in some aspects like patient history, family history, comorbidities, and differential diagnosis. Also, it is important to conduct a detailed interview with the patient caregivers and a physical examination of all body regions [5].

This study aims to present a clinical case of subungual Injury with an initial common presentation and uncommon histopathological diagnosis, a hemangiolymphangioma tumor.

## Case presentation

A 10-year-old female patient came for a consultation, presenting with an injury to the left hallux for several years. She had no personal or family medical history of interest in the direct or indirect relationship of this condition. The condition started with a progressive deformity of the toenail of the left big toe about five years prior to the presentation, according to the patient’s mother. The patient denied pain or changes in the color of the area from the onset of the injury to the day of consultation. Furthermore, she denied previous trauma.

**Figure 1:**
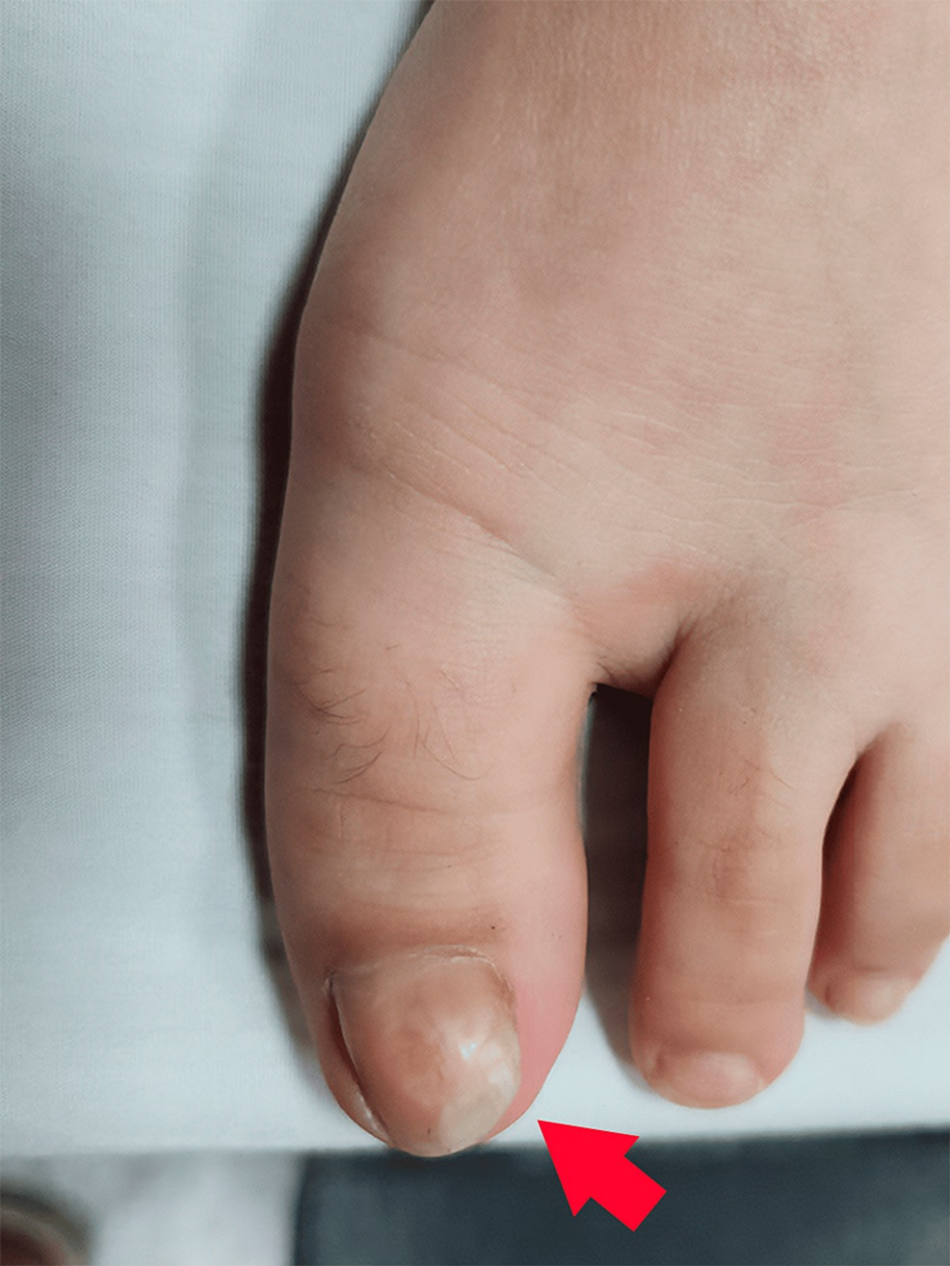
The patient's left big toe revealing an increased nail curvature up to the midline of the nail plate axis, leading to impingement of the midline periungual edge characterizing an onychogryphosis (red arrow).

The reason for consultation was progressive nail deformity (Figure 1), causing some esthetic discomfort to the patient and her family, which was why they decided to see a specialist (dermatologist), who requested auxiliary diagnostic tests (laboratories and imaging) to rule out or confirm an infectious process or glomus tumor. All the laboratory studies were negative, and a soft tissue ultrasound showed a 7-mm irregular bone prominence from the distal phalangeal cortex.

**Figure 2:**
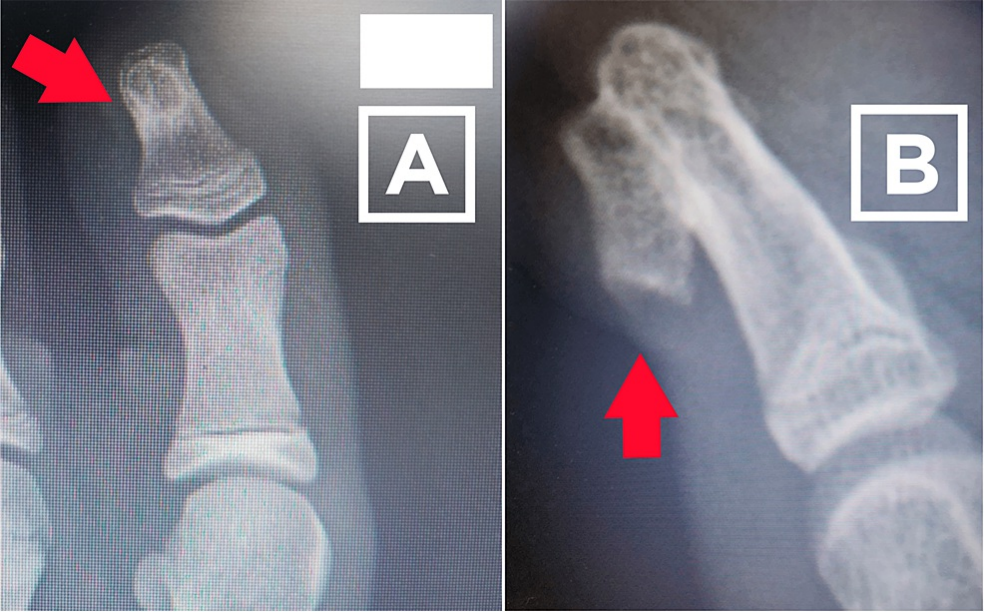
Anteroposterior and lateral X-ray views of the left hallux, showing the injury. (A) Anteroposterior X-ray of the left hallux, where we can observe an sclerotic image by superimposition; (B) Lateral X-ray of the left hallux, where we can observe an exophytic lesion with cortical and medullary continuity with the distal phalanx of the first toe.

Physical examination revealed subungual bone injury to the distal extremity (distal phalanx of the left hallux) (Figure 1), with no other relevant findings. The patient’s pediatrician treated the injury topically with mupirocin with no changes in previous years. To complete the examination requested by the dermatologist, and due to ultrasound findings on soft tissue, the imaging specialist requested an X-ray of the region (Figure 2) and referred the patient to a pediatric orthopedist for better management of the clinical picture.

**Figure 3:**
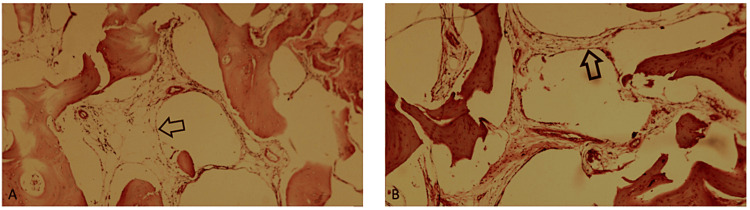
Histopathological image showing intraosseous hemangiolymphangioma (H&E 100x). (A) Dilated vascular structures replacing the bony medullary space. Arrow indicates flat endothelium consistent with lymphatic vessels. (B) Polycystic pattern with several vascular structures delimited by thin, flat endothelium in bone marrow tissue. The arrow in one of the vessels indicates endothelium. This is consistent with lymphatic vascular structure.

Investigation for traumatic injuries, both clinical and through imaging tests (X-ray and soft tissue ultrasound), revealed bone injury. Subungual exostosis was considered as a possible diagnosis, thus prompting the indication for exeresis of the tumoral process. After surgical removal, the resected specimen was sent for pathological assessment, in which it was found that an intraosseous hemangiolymphangioma was the origin of the tumor (Figure 3).

## Discussion

The case of a 10-year-old female patient diagnosed with hemangiolymphangioma was presented. Three months after the operation, no changes were observed in the bone tissue of the patient's distal phalanx, and there were no signs of new growth or recurrence.

The difficulty in classifying this type of lesion in pediatric patients lies in the differential diagnosis which can include tumors such as enchondromas, a benign cartilaginous tumor. Osteochondroma is a benign tumor containing bone and cartilage. Hemangiolymphangioma is a benign tumor with a mixture of blood and lymphatic vessels.

It is essential to conduct a differential diagnosis with other conditions, both benign and malignant, considering the location of the injury. Subungual exostosis is a benign osteocartilaginous tumor that occurs more frequently in the distal phalanges of the toes, and the surgical approach is the gold standard for treatment, with excision or complete curettage being the suggested approach [6]. Regarding osteochondroma, the treatment is surgical by choice, and the approach involves the complete removal of the cartilaginous capsule, including the capsule and the perichondrium surrounding it to prevent recurrences [7].

Hemangiolymphangiomas are known for their benign nature, but due to their potential to invade surrounding structures, a surgical decision is made for their treatment, which involves extensive excision due to suspicion of invasion of adjacent structures. The tumor in this case involved the medullary spaces of the trabecular osseous tissue and was characterized by injuries to the lymphatic vascular lumina of varied shape and size, lined by benign endothelium. Medullary adipose tissue was also observed.

Regarding enchondromas that affect long bones, there is a similarity to our diagnosis since both are radiographic findings, sometimes incidental. However, enchondromas stand out due to the pain associated with the nearby joint or the surrounding soft tissues, in contrast to hemangiolymphangiomas, which typically do not cause pain [8].

We can confidently assert that osteochondroma, similar to the previously described pathologies, requires surgical treatment. Furthermore, when these conditions develop and are surgically addressed during childhood, they tend to recur. In contrast to the conditions mentioned earlier, osteochondroma displays a unique feature in auxiliary diagnostic methods: it presents as a pedunculated lesion resembling cauliflower, with a perichondrium that extends into the periosteum of the underlying bone [9,10].

Subungual exostosis is a benign, slow-growing exostosis located in the toes (or fingers), and it is typically rare in the general population, particularly among pediatric patients. This condition results from a process of bone neoformation involving different stages, and its clinical symptoms depend on its size and associated processes. Subungual exostosis presents as a painful, pinkish, hyperkeratotic tumor of the subungual or periungual region that can lead to onychodystrophy and changes in the surrounding soft tissues. From a histological perspective, it is often surrounded by a fibrocartilaginous layer, with its origin likely linked to prior traumas or infections. Diagnosis is achieved through biopsy, and treatment entails the excision of the lesion from the bone base to prevent recurrences [11,12].

It is worth noting that the prior use of magnetic resonance imaging is described as a means for conducting differential diagnoses. Clinical assessment and focused interviews are crucial for accurate diagnosis and timely surgical decision-making since complete marginal excision of the exostosis appears to minimize recurrence [13-15].

It differs from chronic paronychia, as the latter does not involve bone growth but rather an inflammation of the perionychium (periungual region), which is common in children. Chronic paronychia manifests as a strongly congestive inflammatory reaction in the proximal part of the perionychium, resulting in cuticle loss and alterations in the nail plate in the form of transverse lines or partial or complete changes in nail growth.

Hemangiolymphangiomas are believed to develop as a result of excessive proliferation of vascular formations during the angiogenesis process [16]. Several clinical findings should raise suspicion of this diagnosis in childhood, such as onychogryphosis (thickened nails) or pincer nail (Figure 1), a dystrophy resulting from multiple causes characterized by a pincer-shaped nail plate, pain, and often exostosis of the surrounding phalanx. There is an increase in the transverse curvature of the nail along the longitudinal axis of the nail plate, leading to impingement of the periungual edges (crawling nails) [17].

Vascular malformations are anomalies caused by disruptions in vasculogenesis. Depending on the histological structure predominantly present, they can consist of various combinations of vascular elements and are termed hemangiolymphangioma or lymphangiohemangioma. Hemangiolymphangioma can occur in multiple anatomical sites, including the head and neck, axilla, abdominal cavity, extremities, urinary bladder, and oral cavity, among others [18]. While it is a benign tumor, it still has the potential to invade organs. Surgery is regarded as the most effective treatment, and the excision should encompass the surrounding tissue suspected of being invaded [19]. This condition represents a rare developmental anomaly, and a definitive diagnosis is achieved through histopathological examination. Long-term follow-up of the patient is recommended to rule out recurrences [20].

## Conclusions

This study presented a clinical case whose final diagnosis (intraosseous hemangiolymphangioma) was classified as infrequent in terms of location, age, and type of presentation.

Hemangiolymphangiomas, which are rare vascular anomalies, require recognition because of their difficult diagnosis. It is essential to consider them when assessing vascular anomalies and to be aware of the various treatment options available to ensure an effective approach and a favourable prognosis for the patient.
